# Life expectancy versus lifespan inequality: A smudge or a clear relationship?

**DOI:** 10.1371/journal.pone.0185702

**Published:** 2017-09-28

**Authors:** László Németh

**Affiliations:** Laboratory of Survival and Longevity, Max Planck Institute for Demographic Research, Rostock, Germany; Universidad Veracruzana, MEXICO

## Abstract

Interest in inequality, including lifespan inequality, is growing. Several studies, using various measures of variation in the length of life, reveal that as life expectancy increases, lifespan inequality tends to decrease, albeit with considerable variation across populations and over time. The aim of this article is to understand why the strength of the relationship between life expectancy and lifespan inequality varies across publications. Results differ in large part because they are based on different data sources. In addition, some measures show more smudginess than others. All the analyses presented here support the basic finding of a strong relationship between life expectancy and lifespan inequality.

## Introduction

As life expectancy rises [1], how is its relationship with lifespan inequality changing? The strong negative correlation between measures of lifespan inequality, i.e., discrepancies in how long individuals live, and life expectancy is well known [2, 3]. Several studies, using various measures of variation in the length of life, have explored aspects of this [4–10]. These studies reveal that as life expectancy increases, lifespan inequality tends to decrease, albeit with considerable variation across populations and over time.

Some of the analyses of life expectancy vs. life inequality make use of many life tables from these and other sources. For instance, Smits and Monden [5] present a scatterplot for adult populations, aged 15+, covering males and females over many years of time in 212 countries. Vaupel et al. [8] do so for 7056 life tables in the HMD. Hence, this article presents graphs that include a wide range of life tables.

The aim of this article is to understand why the strength of the relationship between life expectancy and lifespan inequality varies across publications. Is this variation mainly due to the alternative measures used? Or is it mainly attributable to differences in the populations considered?

## Methods

This study is based on period life tables available in the Human Mortality Database (HMD) [11], all life tables available in the Human Life-Table Database (HLD) [12], life tables for periods between 1950 and 2015 estimated in the 2015 Revision of World Population Prospects (WPP) [13], and life tables provided by the World Health Organization (WHO) [14]. These sources contain the most frequently used life tables because of their availability, coverage and documentation.

The HMD database contains carefully checked life tables that have been compiled using strict standards and similar procedures. Vital statistics provide raw data, birth and death counts, while population counts are derived from periodic censuses or official population estimates. Sources of raw data and exact methodology of specific adjustments for each population are well documented [15]. The WHO database includes life tables that have been compiled using comparable methods developed by WHO but some of these life tables are based on scarce or problematic data [16]. The WPP database is a collection of life tables estimated by the Population Division of the United Nations; as for the WHO life tables, some of these life tables are based on poor-quality data [17]. The HLD database contains a rather miscellaneous collection of life tables from various sources and of varying quality [18].

Several alternative measures have been used to capture aspects of lifespan equality and inequality.

1. One is life expectancy lost due to death, also called life disparity and denoted by *e*^†^ [8]. At birth it is defined by $e_{0}^{†}=\int_{0}^{\omega }e(x)f(x)dx$, where $e(a)=\int_{a}^{\omega }l(x)dx/l(a)$ is remaining life expectancy at age *a*, $l(a)=exp(-\int_{0}^{a}\mu (x)dx)$ gives the probability of survival to age *a* and *μ*(*a*) denotes the age-specific hazard of death. The life table distribution of deaths is given by *f*(*a*) = *l*(*a*)*μ*(*a*). Maximum lifespan is denoted by *ω*. This measure was used by van Raalte et al. [7], Vaupel et al. [8] and van Raalte et al. [10].
2. A second measure is the Gini coefficient. It can be calculated from birth or from a later age such as age 15. The Gini coefficient of lifespans from birth is defined by $G_{0}=1-\int_{0}^{\infty }{\left[l\left(x\right)\right]}^{2}\text{d}x/e\left(0\right)$. See [19] for further discussion. The Gini coefficient was used by Smits and Monden [5].
3. A third measure is the entropy of the life table, which Keyfitz [20] defined as $H=-\int_{0}^{\infty }\text{ln}(l(x))l(x)dx/\int_{0}^{\infty }l(x)dx$. It can also be defined, equivalently, by $H=e_{0}^{†}/e(0)$ [21]. It was used by Noymer and Coleman [22].
4. The coefficient of variation, defined as the standard deviation of age at death divided by the mean age at death, is a fourth measure that is sometimes used. For instance, Edwards and Tuljapurkar [4], Engelman et al. [6] and Gillespie et al [9] calculate the coefficient of variation of lifespans above various ages, including ages 10, 15, 50 and 75.

Wilmoth and Horiuchi [2] showed that most frequently used indicators of lifespan inequality, the Gini coefficient, Keyfitz’s entropy and the coefficient of variation, are highly correlated with each other; Vaupel et al. [8] showed that life expectancy lost due to death is also highly correlated with the three other measures.

## Results

**Fig 1:**
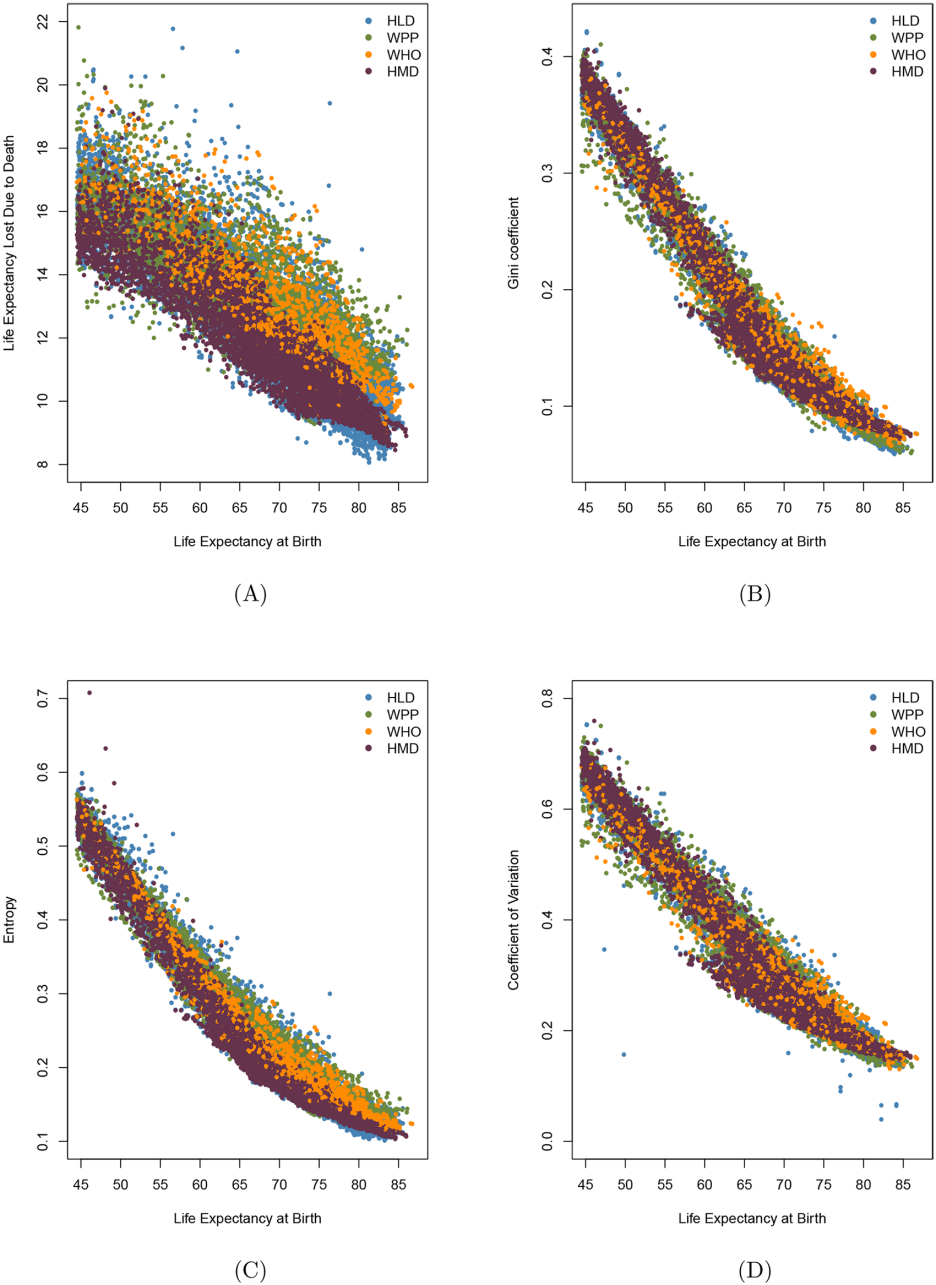
Life expectancy at birth and life inequality measures for all the life tables with greater than 45 years of life expectancy from Human Mortality Database (HMD), Human Life-table Database (HLD), the World Population Prospects (WPP) and World Health Organization (WHO) databases. A: life expectancy at birth (*e*_0_) vs. life expectancy lost due to death (*e*^†^), B: *e*_0_ vs. Gini coefficient (*G*_0_), C: *e*_0_ vs. Keyfitz’s entropy (*H*), D: *e*_0_ vs. coefficient of variation.

Fig 1 presents the relationship between life expectanccy at birth and the four main measures of lifespan inequality: life expectancy lost due to death (Fig 1A), the Gini coefficient (Fig 1B), Keyfitz’s entropy (Fig 1C) and the coefficient of variation (Fig 1D). In all four cases measures calculated from HLD data show the highest variation, as might be expected given the miscellaneous nature of this database. Measures calculated from WPP data show almost as high variation, probably because some of these life tables pertain to populations with data of questionable quality. Because points in the Figures overlap, a list of all the points calculated from HMD and WHO databases was created and then points were chosen at random, without replacement, and plotted on top of the points corresponding to HLD and WPP data. Note in comparing Fig 1A, 1B, 1C, and 1D that the smudge is broadest for life expectancy lost due to death and narrowest for Keyfitz’s entropy.

Wrycza et al. [23] suggest Gini coefficient, Keyfitz’s entropy and the coefficient of variation as preferable measures of shape within the pace shape framework introduced by Baudisch [24]. This could explain why the smudge in Fig 1A is much wider than in B, C and D: because *e*^†^ is not a shape measure, as it depends on units of time (remaining life years left), whereas the other measures are scaled by life expectancy. Missov et al. [25] compare life expectancy values in life tables based on the methods applied by the highest quality life-table databases. Life tables published in HMD, WHO and WPP databases use smoothing techniques for mortality rates at oldest-old ages in order to address right censoring adequately. HLD, in contrast, contains original life tables and this could shed light on why points corresponding to HLD are the most dispersed.

**Fig 2:**
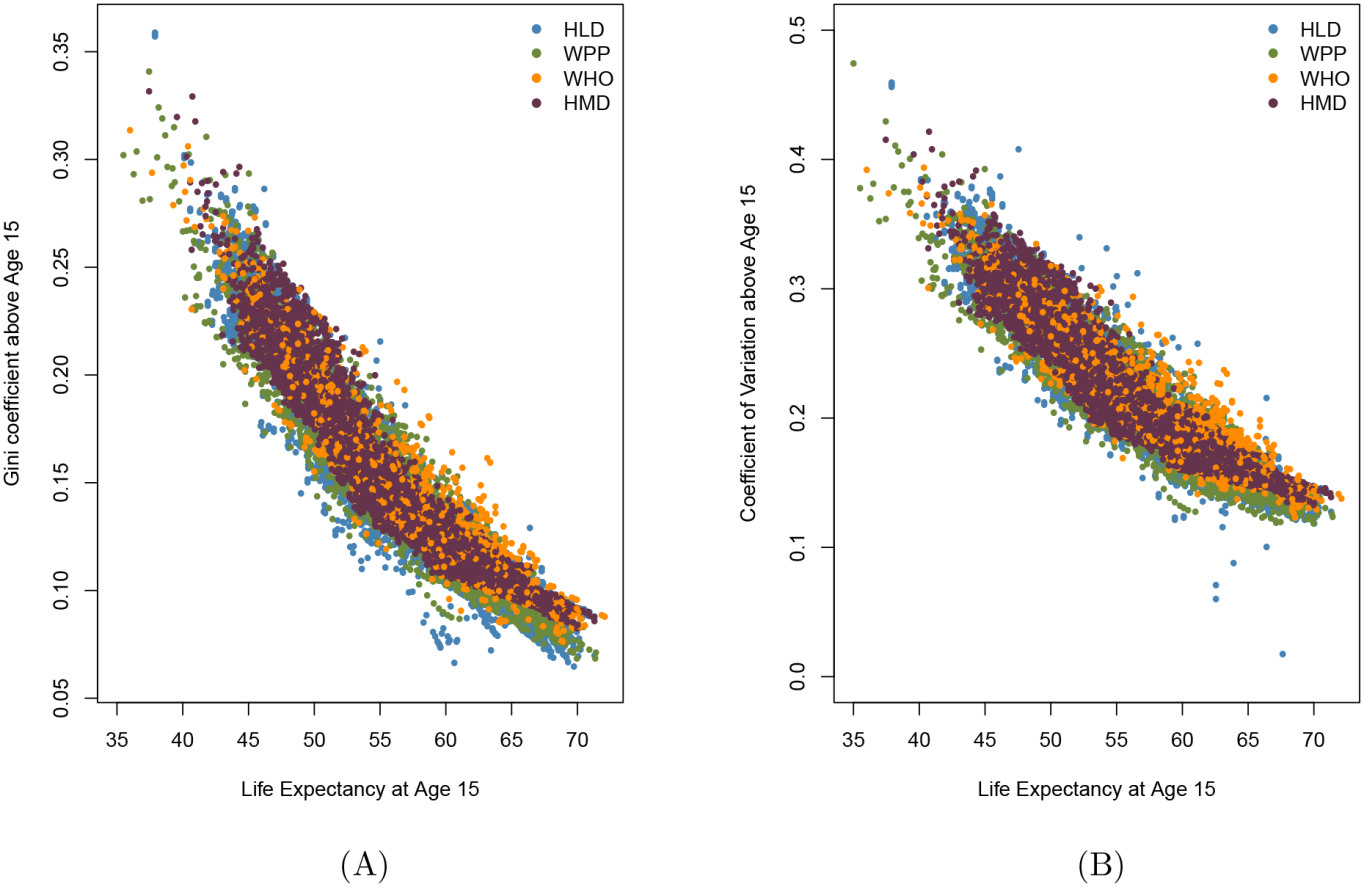
Life expectancy at age 15 and life inequality measures for adult populations (aged 15+) from Human Mortality Database (HMD), Human Life-table Database (HLD), the World Population Prospects (WPP) and World Health Organization (WHO) databases. A: Life expectancy at age 15 (*e*_15_) vs. Gini coefficient above age 15 (*G*_15_), B: life expectancy at age 15 (*e*_15_) vs. coefficient of variation above age 15 (*cv*_15_).

Several publications present life expectancy and a measure of lifespan inequality not from birth but from a later age. For example, Smits and Monden [5] present a comparison of life expectancy and the Gini coefficient from age 15, the “adult” population. Edwards and Tuljapurkar [4] and Gillespie et al. [9] argue that comparisons of life expectancy and lifespan inequality are cleaner if the coefficient of variation of lifespans is calculated after excluding infant and childhood mortality. Fig 2A plots life expectancy at age 15 against the Gini coefficient above this age; Fig 2B does so against the coefficient of variation above age 15. Comparison of Fig 2A with the similar Figs 1B and 2B with the similar Fig 1D suggests that the patterns may not be fundamentally different or less messy if the analysis is restricted to ages 15+ as opposed to including all individuals from birth.

**Fig 3:**
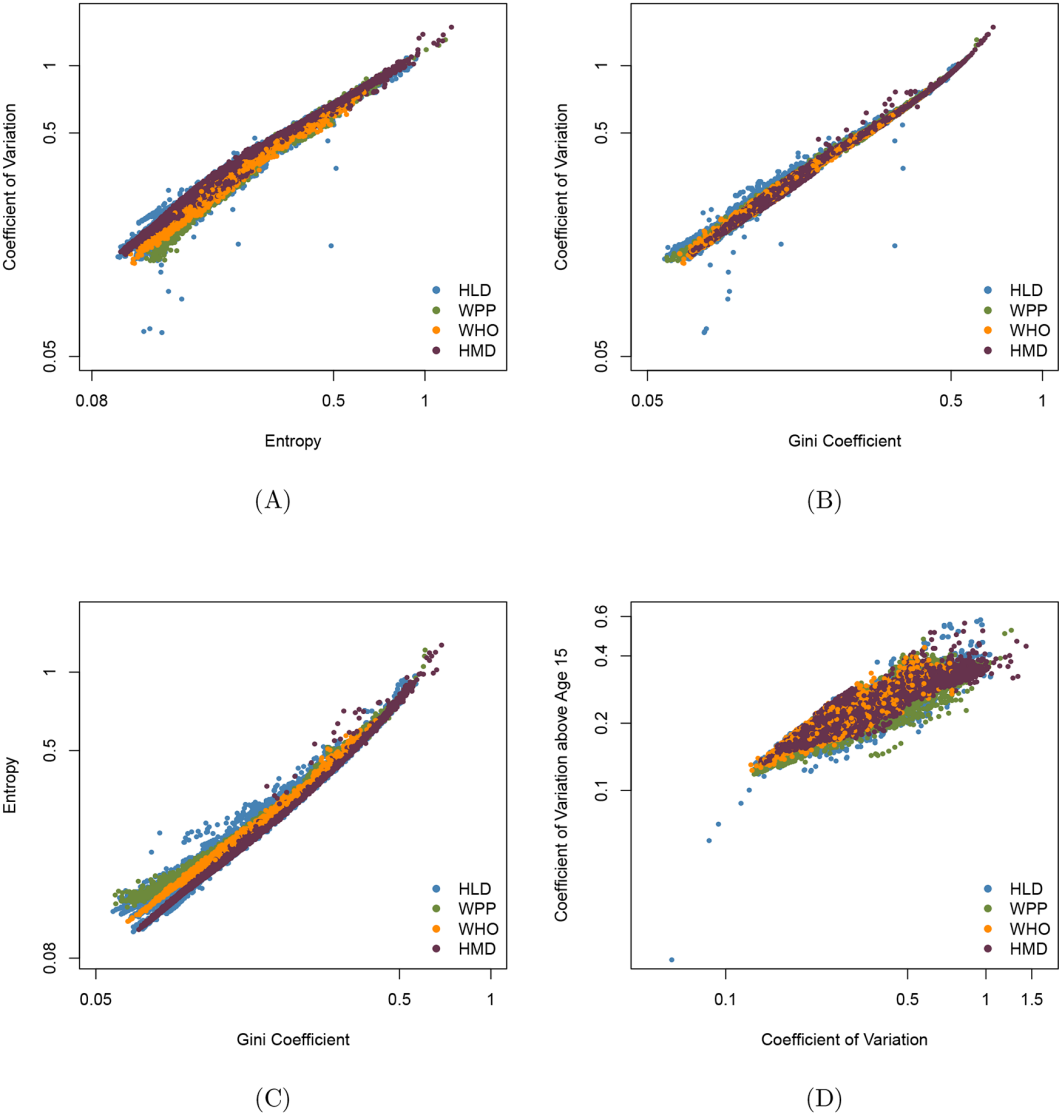
Relationships between lifespan inequality measures (on a log scale) for Human Mortality Database (HMD), Human Life-table Database (HLD), the World Population Prospects (WPP) and World Health Organization (WHO) databases. The corresponding Pearson correlation coefficients are A: *ρ* = 0.976, B: *ρ* = 0.992, C: *ρ* = 0.990, D: *ρ* = 0.944.

Fig 3 sheds further light on the consequences of using one measure of lifespan inequality vs. another. It can be seen that Keyfitz’s entropy, the Gini coefficient and the coefficient of variation at birth and at age 15 are highly correlated. Note however that Fig 3A and 3B indicate that the coefficient of variation sometimes falls well below the value that might be expected from a regression of this measure on either Keyfitz’s entropy or the Gini coefficient. This suggests that it would be judicious to check analyses based on the coefficient of variation with corresponding analyses based on Keyfitz’s entropy or the Gini coefficient.

**Fig 4:**
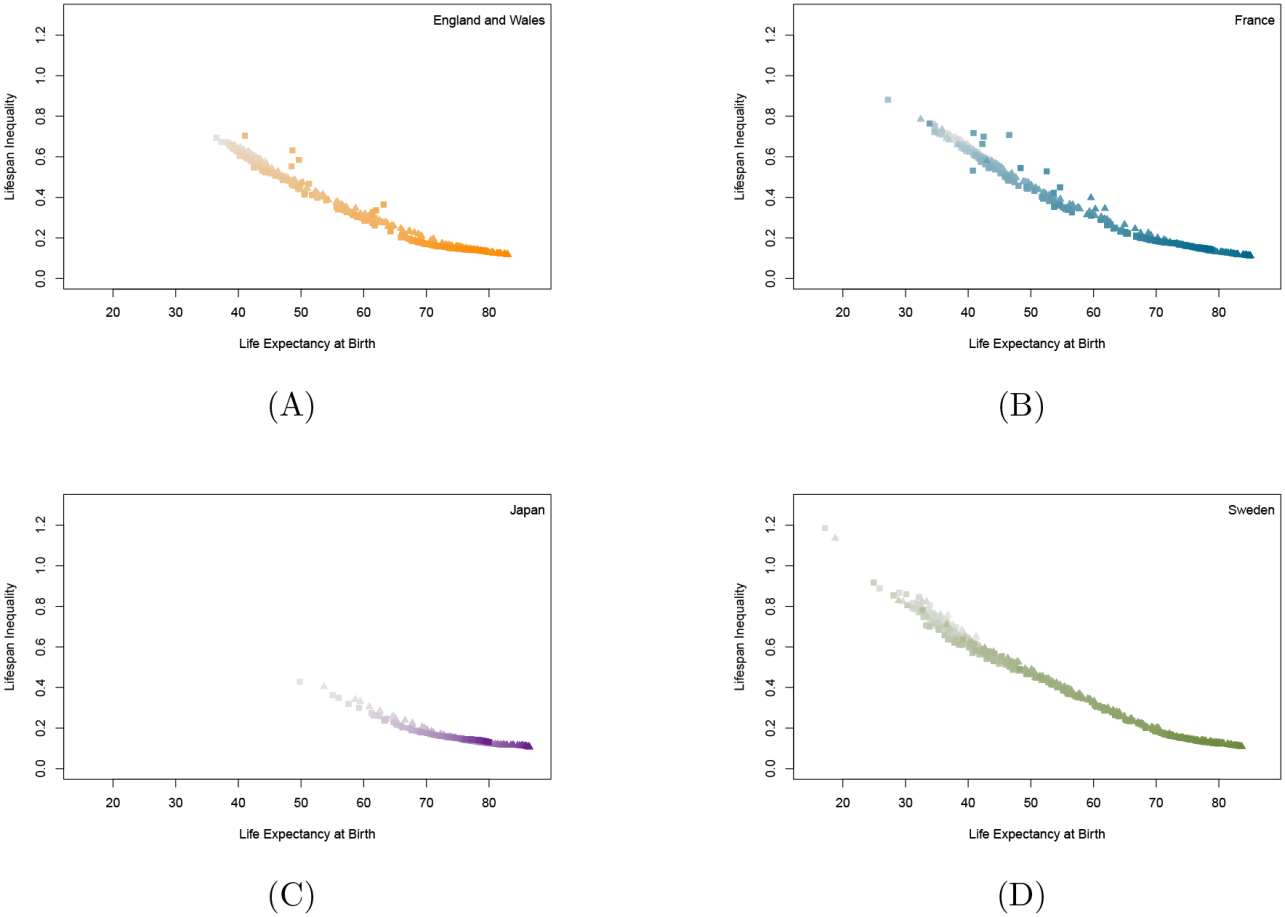
Female and male lifespan equality vs. life expectancy for selected countries. Females are denoted by triangles, males by squares. More recent values are designated with stronger colors. A: England and Wales in 1841–2013, B: France in 1816–2013, C: Japan in 1947–2012, D: Sweden in 1751–2014.

Finally, to more deeply understand patterns within specific countries, HMD data was used to produce graphs for three countries—England and Wales, France and Sweden—for which many years of data are available as well as for Japan, which is the world’s leader in female life expectancy. These graphs, shown in Fig 4, show a near-linear relationship between life expectancy at birth and lifespan inequality at birth, as measured by Keyfitz’s entropy, although with subtle differences from country to country. The four patterns are far cleaner than the patterns shown in Figs 1, 2 and 3, indicating that much of the smudginess in those figures is due to differences among populations in large collections of diverse populations, some with data of poor quality. Colchero et al. [26] revealed a strikingly linear relationship between life expectancy and lifespan equality measures for these many other contemporary and historical populations.

## Conclusion

This article explores why results in some publications show relatively high variation while others reveal a more linear relationship between life expectancy and measures of lifespan inequality. Based on the most commonly used life-table databases it can be concluded that results in publications differ in part because they rely on different data sources.

The HMD database only includes data from those countries and time periods with reliable compilations of vital statistics; these data are carefully checked and then consistently processed by standardized methods. In contrast, both the WHO and WPP databases include life tables for almost all the world’s countries; in many cases, the available vital statistics data are sparse or problematic. The HLD is a jumble of miscellaneous life tables from various sources, compiled from data of varying quality and estimated using various methods. Both the varying quality of the data in the four databases and the different methods used to estimate life tables [25] influence the scatter of points shown in the four Figures.

In addition, some measures (e.g., life expectancy lost due to death) show more smudginess than others, probably due to differences in scaling [23]. Hence three factors—the use of widely different data sources, the application of different methods for estimating life tables, and variation in how tightly a measure of life span inequality is associated with life expectancy—influence the degree of smudginess shown in the Figures. Understanding the relative impact of these three factors requires further analysis.

The study suggests that the chosen life inequality measure, the chosen countries and the inclusion or exclusion of younger ages make graphs more or less messy but that all the analyses support the basic finding of a strong relationship between life expectancy and lifespan equality or inequality. This important relationship seems to be valid even for primate and historical human populations as well [26]. Given this relationship, it may be possible (and useful) to forecast both at the same time.
